# Les fractures pathologiques secondaires des tumeurs bénignes chez l'enfant: à propos de 20 cas

**DOI:** 10.11604/pamj.2015.21.204.6556

**Published:** 2015-07-20

**Authors:** Youssef Nader, Idrissi Khalid Koulali, Salahi Hicham

**Affiliations:** 1 Pôle de traumatologie et orthopédie, Hôpital Militaire d'Instruction Avicenne, Université Kadi Iyade, Faculté de Médecine et de Pharmacie, Marrakech, Maroc

**Keywords:** Fractures pathologiques, tumeurs bénignes, enfant, pathological fractures, benign tumors, infant

## Abstract

Une fracture est dite pathologique ou sur os pathologique quand elle survient sur un tissu osseux remanié par un processus pathologique, elle ne tient compte, ni du mécanisme de la fracture ni de la lésion préexistante, on élimine ainsi les fractures dites de fatigue, survenant sur un os normal. Elles constituent un événement assez rare en traumatologie pédiatrique. Ces traumatismes posent au praticien un double problème, celui de la fracture et celui de la pathologie tumorale en cause. Le but de notre étude est de décrire le profil de ces fractures et d'analyser leur prise en charge dans notre contexte. Nous avons réalisé une étude rétrospective sur une période de 10 ans au service. Nous avons colligé 20 cas. L’âge moyen était de 10 ans, 70% étaient de sexe masculin. 98% des traumatismes étaient de faible énergie. 80% des lésions ont touché soit le fémur soit l'humérus, le kyste osseux essentiel était la principale étiologie causale. Le résultat final était bon dans 60% des cas. La prise en charge de ces fractures dépend surtout de la tumeur causale et des caractéristiques de la fracture. Le rôle de la radiologie est incontournable et let traitement doit se faire selon une méthodologie précise.

## Introduction

Les fractures pathologiques chez l'enfant sont toujours d'actualité, la découverte de telles lésions pose un certain nombre de problèmes aussi bien pour l'urgentiste que le spécialiste, par la multiplicité des étiologies qui les engendrent et surtout les difficultés thérapeutiques qu'elles posent. Nous avons consacré ce travail aux lésions osseuses bénignes à savoir, les tumeurs Bénignes, les pseudotumeurs sauf les infections osseuses. Toutes ces lésions tumorales ont des points communs dans la gestion et la prise en charge thérapeutique. Le but de ce travail et de connaître le profil des ces fractures dans notre contexte et de décrire la stratégie de prise en charge en analysant une série rétrospective et une revue de la littérature.

## Méthodes

Nous avons réalisé une étude rétrospective de 20 cas concernant les fractures sur os pathologique chez l'enfant. Ce travail a été réalisé au sein de notre service, et ce durant la période allant de janvier 1993 à janvier 2013. Nous avons défini les critères d'inclusions suivants: un recul minimum de 18 mois, une fracture sur os à cartilage de croissance encore ouvert au moment du diagnostic, une fracture pour laquelle la prise en charge a été entièrement réalisée par l’équipe du service, les fractures compliquant les lésions tumorales bénignes. Nous avons défini les critères d'exclusions suivants: les fractures survenues sur une ostéogenèse imparfaite ou autre fragilité osseuse acquise ou constitutionnelle, les fractures compliquant les tumeurs osseuses malignes ou autres éthologies infectieuses, les fractures pathologiques pour les quelles le diagnostique histologique n'est pas précisé. Pour chaque patient, nous avons recueilli les informations suivantes sous formes d'une fiche d'exploitation, résumé sur (Tableau 1), placé en fin du texte.

**Table 1:** Demographic characteristics of the study participants

Variables	Frequency	Percent
**Participants**		
Limpopo	212	28.3%
KwaZulu-Natal	220	29.4%
Eastern Cape	316	42.2%
**Gender**		
Male	296	37.9%
Female	486	62.1%
**Age group**		
Less than 20 years	59	7.5%
20-29 years	292	37.3%
30-39 years	163	20.8%
40-49 years	124	15.9%
50-59 years	83	10.6%
60 years and older	61	7.8%
**Marital status**		
Single	574	73.4%
Married	164	21%
Divorced	16	2%
Widowed	28	3.6%
**Racial group**		
African	767	98.1%
White, Coloured and Indian	16	1.9%
**Employment status**		
Employed	191	24.4%
Unemployed	591	75.6%
**Educational level**		
No education	31	4%
Primary education	116	14.8%
Grade 10	239	30.6%
Grade 12	303	38.7%
Tertiary	64	8.2%
Other post-school education	29	3.7%

## Résultats

Dans cette étude, 20 patients ont répandu à nos critères d'inclusion, qui résume le profil de nos observations sur le (Tableau 2), placé en fin du texte: l’âge moyen des patients était de 10 ans avec des extrêmes de 5 et 15 ans. Nous relevons 14 cas de sexe masculin (70%) et 6 cas de sexe féminin (30%). Les circonstances de survenue de la fracture pathologique étaient: par chutes: 14 cas (70%), par traumatismes: 4 cas 20%, spontanées: 1 cas (5%) et 1cas non précisée 5%. L’énergie du traumatisme était: haut énergie chez 2 patients soit (10%) et de faible énergie chez 18 patients soit (90%). Le siège de la fracture étaient métaphysaires chez 15 patients soit (75%) et diaphysaires chez 5 patients soit (25%). L'os concerné par la fracture: Le fémur a été touché dans 10 cas 50%, l'humérus dans 7 cas (35%), le tibia dans 2 cas soit (10%) et la fibula dans 1 cas soit (5%). Le Type de fracture: la fracture était complète dans 6 cas (30%), en bois vert dans 10 cas 50% et en motte de beurre dans 4 cas soit (20%). Les pathologies responsables des fractures pathologiques dans notre série étaient: Le kyste osseux essentiel chez 12 enfants (60%), le kyste osseux anévrysmal chez 3 enfants (15%) le fibrome non ossifiant chez 3 enfants, (15%) l'ostéoblastome chez un enfant (5%), le chondrome solitaire chez un enfant (5%). Le traitement était orthopédique dans 7 cas (35%): ce traitement a concerné 7 KOE (4 fémurs, 2 humérus, 1 tibia). Le traitement a été chirurgical chez 13 enfants (65%): ce traitement a concerné 5 KOE (3 fémurs et 2 humérus), les 3 KOA, 1 OBM et les 2 FNO. les résultats suite aux différents traitements: pour les **Kystes osseux essentiels** traités orthopédiquement, 5 ont eu un bon résultat (3 fémurs, 1 humérus et un tibia); un humérus a eu un résultat moyen et un fémur a eu un mauvais résultat, ceux opérés 6 bons résultats ont été obtenus dont 3 fémurs traités par curetage+greffe et lame plaque, 2 humérus traité par curetage-greffe et ECMES et un tibia traité par curetage-greffe. Un seul résultat a été jugé moyen au niveau d'un fémur proximal traité par curetage, greffe et lame plaque, et 2 mauvais résultats ont succédé à 2 fémurs greffés, vissé et à 1 humérus curetés et greffés. Pour le **kyste osseux anévrysmal**: tous opérés par curetage-greffe sans ostéosynthèse pour le tibia avec un bon résultat et avec ostéosynthèse pour le fémur et l'humérus avec des résultats moyens. Pour **le fibrome non ossifiant**: tous opérés. Les résultats étaient bons au niveau de fémur et humérus avec curetage greffe et ostéosynthèse et moyen au niveau de la fibula avec résection. Le recul: le délai de recul moyen était de 24 mois avec un minimum de 18 mois et un maximum de 96 mois. Les complications: 16 cas sont guéris sans complications, 4 cas kystes osseux essentiel ont présentés des complications. Les Résultats finaux étaient bons dans 11 cas (55%), moyens dans 5 cas (25%) et mauvais dans 4 cas (20%).

**Tableau 2:** Profil de nos observations

Age	Sexe	Circonstances	Energie	Os	Siège	Type	Anapath	Traitement	Résultat
9	M	chute	faible	fémur	métaph	C	KOE	Orthopédique	mauvais
5	M	traumatisme	faible	humérus	diaphy	C	KOE	Orthopédique	bon
12	F	chute	faible	fémur	métaph	C	KOE	Orthopédique	bon
8	M	traumatisme	haute	fémur	métaph	BV	KOE	Orthopédique	bon
8	F	chute	faible	fémur	diaphy	C	KOE	Orthopédique	bon
11	M	chute	faible	humérus	métaph	MB	KOE	Orthopédique	bon
15	M	chute	faible	fémur	métaph	BV	KOE	Orthopédique	bon
8	M	traumatisme	faible	fémur	métaph	BV	KOE	Curetage+VIS	mauvais
14	M	chute	faible	humérus	métaph	C	FNO	Curetage+Greffe	bon
13	M	chute	faible	fémur	métaph	C	KOA	Curetage+Greffe	bon
15	M	chute	faible	fibula	métaph	BV	FNO	Résection	moyen
11	F	chute	faible	humérus	métaph	MB	OBM	Ablation nidus +greffe	moyen
9	M	chute	faible	humérus	métaph	BV	KOE	Curetage+ Greffe+ECMS	bon
13	F	spontanée	faible	tibia	métaph	BV	KOE	Curetage+ Greffe+ECMS	bon
5	M	chute	faible	fémur	diaphy	C	FNO	Curetage+ Greffe+ECMS	bon
13	M	chute	faible	humérus	diaphy	BV	KOA	Curetage+ Greffe+ECMS	moyen
12	F	chute	faible	fémur	métaph	MB	KOE	Curetage+ Greffe+ Lame plaque	bon
10	M	chute	faible	fémur	métaph	C	KOE	Curetage+ Greffe+ Lame plaque	bon
6	F	Non précisée	haute	tibia	métaph	BV	KOA	Curetage+ Greffe+ Lame plaque	bon
7	M	traumatisme	faible	fémur	diaphy	MB	CS	Réduction+ Greffe+ ECMS	bon

**M**:masculin **F**:FEMININ, **C**:COMPLETE, **BV**:BOIT VERT; **MB**:MOTTE DE BEURRE. **KOE**:KYSTE OSSEUX ESSENTIEL. **KOA**:KYSTE OSSEUX ANEVRYSMALE. **FNO**:FIBROME NON OSSIFIANT. **CS**: CHONDROME SOLITAIRE. **OBM**:OSTEOBLASTOME. **ECMS**: ENCLOUAGE CENTROMEDULLAIRE SOUPLE.

## Discussion

**Figure 1:**
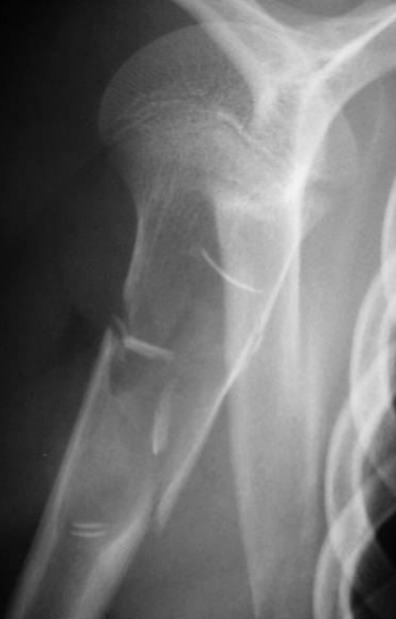
Fractures métaphysaire sur kyste osseux éssentiel, de l'humérus

**Figure 2:**
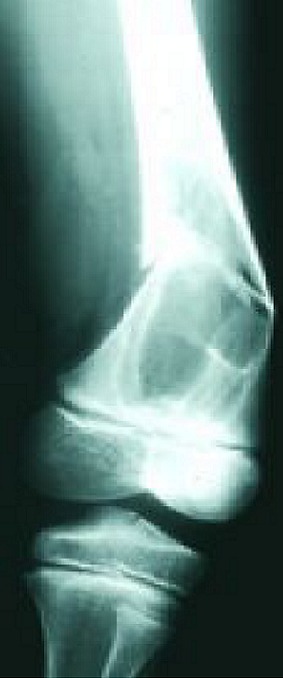
Fracture métaphysaire inferieure sur kyste osseux anévrysmal du femur

**Figure 3:**
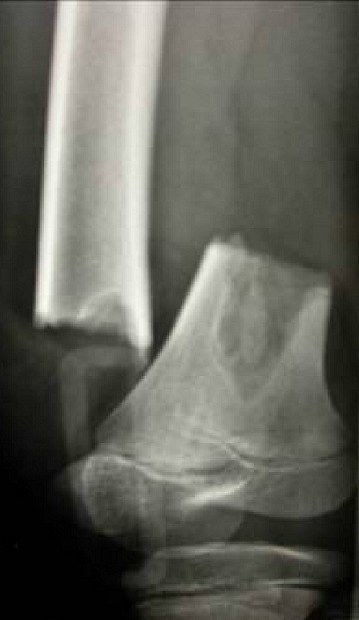
Fracture métaphysaire inferieure sur fibrome non ossifiant du femur

Les fractures pathologiques secondaires aux tumeurs osseuses bénignes chez l'enfant constituent un événement assez rare en traumatologie pédiatrique, Au niveau du notre série, 20 cas ont été retenus en 10 ans. Notre série comporte un échantillon assez réduit par rapport à la série de Datir et al [1] et à celle d'Ortiz [2], ceci est probablement expliqué par nos critères de sélection. Le profil épidémiologique de ces fractures pathologiques montre que: la prédominance masculine est toujours notée: 57% dans la série de Ortiz et al [2], 59% dans la série de Datir et al [1] et 70% dans la notre. L’âge de survenue de ces fractures: rejoint celui des fractures non pathologiques. La moyenne se situe entre 9 et 10 ans. Dans notre série, la moyenne d’âge était de 10 ans. L’énergie du traumatisme causal: Tous les auteurs s'accordent pour dire que le mécanisme causant ce type de fractures est un traumatisme de faible énergie [3] et [4]. Chez nos 20 patients (90%) des fractures ont succédé à un traumatisme de faible énergie. Les Principales étiologies tumorales bénignes dans notre série sont: **Le kyste osseux essentiel (KOE)**: est de loin le plus fréquent de ces causes. **Le kyste osseux anévrysmal (KOA), le fibrome non ossifiant (FNO), ostéochondromes (OCH) et les chondromes (CH)** donnent également des fractures mais à des proportions variables. Les localisations des ces fractures dépendant essentiellement de la pathologie causale: Dans notre série, le siège métaphysaire a été concerné dans plus des 2/3 des cas (67%). Ce constat a été rapporté aussi par Ortiz et al [2] avec 70% de localisation métaphysaire. A notre avis, cette prédominance pourrait être expliquée par le faite que la région métaphysaire est un tissu spongieux mécaniquement moins résistant que l'os diaphysaire, de toutes étiologies confondues, le fémur, l'humérus et le tibia partagent les localisations de ces fractures sur le squelette. Dans la série de Datir et al [1] et celle d'Ortiz et al [2], l'humérus a été touché dans 40% et le fémur dans 30% des cas. Des résultats proches ont été retrouvés dans notre série. Le risque de survenue de fracture est considérablement conditionné par le stade d'agressivité de ces tumeurs osseuses bénignes, Dormans et al [4] distinguent 3 stades: **Le stade I**: lésions bénignes asymptomatiques, exceptionnellement compliquées de fractures. **Le stade II** correspond à des lésions dites intermédiaires. **Le stade III** est représenté par les lésions agressives, symptomatiques et à croissance rapide où le risque fracturaire est important. Le profil de ces fractures pathologiques varie en fonction de l’étiologie: Les fractures sur Le **(KOE)** (Figure 1): Environ 75% des kystes osseux de l'enfant sont découverts lors d′une fracture [5]; peu déplacées, consolidant dans des délais normaux. En revanche, le kyste ne guérit au cours de cette consolidation que dans 10% à 30% des cas [5]. La plupart des auteurs [6–8] estiment que le risque de fracture est d'autant plus important lorsque: Le kyste est actif, douloureux et augmente de taille sur les radiographies successives. Dans notre série, 10 des 12 kystes osseux essentiel compliqués de fractures étaient actifs (83%). Les fractures sur le **(KOA)** (Figure 2): environ 10 à 35% des kystes anévrismaux sont découverts à l′occasion d′une fracture [9, 10], si la fracture consolide de manière habituelle, le kyste anévrismal continue à évoluer dans la majorité des cas et une nouvelle fracture survient. il faut donc traiter la lésion de manière spécifique. La lésion est souvent agressive et expansive qu'elle soit centrale ou excentrique. Nos 3 kystes osseux anévrysmal correspondaient à cette description. Les fractures sur le **(FNO)** (Figure 3): habituellement, il ne devient symptomatique qu'en cas de fracture. Dans la littérature, prés de 45% des fractures sur ce type de tumeur intéressent le tibia distal et la grande majorité de ces fractures surviennent lorsque la tumeur dépasse 3-4 cm longueur et surtout lorsqu'elle occupe plus que 50% de la largeur osseuse. Dans notre série, 3 cas ce sont compliqués de fracture étaient des lésions larges, le potentiel de consolidation de ces fractures est excellent [11, 12]. Le risque de fracture itérative, bien que présent est faible [12]. Les fractures sur les **(OCH)**: ces lésions sont les plus fréquentes des tumeurs osseuses bénignes de l'enfant et ne sont symptomatiques que lorsqu'ils rentrent en conflit avec les structures de voisinage [4]: telle La paralysie du sciatique poplité externe peut survenir en association par exemple avec un ostéochondrome fibulaire proximal [13]. Les fractures sur ostéochondrome sont rares. Nous avons rapporté un seul cas d'exostose solitaire pédiculée compliquée de fracture chez un enfant de 7 ans. Les fractures sur les **(CH)** [4].

**Figure 4:**
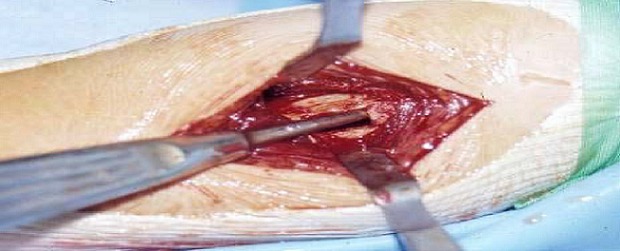
Curetage associé à une greffe osseuse du kyste essentiel humeral

La forme solitaire est rare chez l'enfant mais la survenue de fracture est plutôt fréquente, principalement au niveau des os tubulaires de la main et du pied. A l'opposé, l'enchondromatose multiple ou la maladie d'Ollier est plus présente chez l'enfant entre 2 et 10 ans. Des fractures ont été rapportées dans ce contexte, nous n'avons pas noté cette situation dans notre série. Les fractures sur **Dysplasie fibreuse (DF)**: c'est une tumeur fibreuse expansive pouvant se présenter sous trois formes, toutes entrainent des déformations et des fractures dans 40%. Nous n'avons pas noté cette situation dans notre série. Les fractures sur **granulome éosinophile (GE)** [4] tumeur osseuse bénigne ostéolytique, telle L'histiocytose X est la principale tumeur osseuse bénigne avant l’âge de 4ans. L'incidence de fractures est d'environ 14%. Nous n'avons pas noté cette situation dans notre série. Les fractures sur **La tumeur à cellules géante (TCG)** [14]. Cette tumeur est très rare avant fermeture du cartilage de croissance. Le fémur distal, le tibia proximal et l'humérus proximal sont les principales localisations. Les fractures surviennent en moyenne chez 16% des patients. Nous n'avons pas noté cette situation dans notre série. La gestion pratique de ces fractures pathologiques: commence par l'immobilisation immédiate, provisoire et systématique du foyer de fracture, tout enfant victime d'une fracture doit bénéficier d'un examen clinique général le jour de son admission, après analyse des éléments sémiologiques rapportés par les radiographies simples, les hypothèses diagnostiques sont émises et permettent d'orienter les examens complémentaires. Plusieurs situations peuvent se présenter: La lésion est bénigne, de diagnostic évident, dans ce cas aucune imagerie complémentaire n'est nécessaire, ou la fracture révèle une lésion peu agressive, d’évolution lente pour laquelle le diagnostic radiologique est hautement probable et le traitement chirurgical de la lésion est nécessaire, dans ce cas, une TDM ou une IRM est souhaitable pour une meilleure localisation de la lésion avant la biopsie qui devient systématique devant une lésion très agressive de grande taille faisant suspecter un processus malin et chaque fois qu'il y'a « un doute». Elle doit être planifiée techniquement en fonction des données de l'imagerie. Le traitement dépend avant tout de la nature de la lésion responsable de la fracture et ensuite du type fracturaire [15, 16], Dormans et al [4, 17] a proposé de classer le traitement des fractures pathologiques en 3 groupes: **Type 1**: abstention thérapeutique sur la tumeur par traitement orthopédique de la fracture: ce groupe concerne surtout les fractures sur FNO et Certains KOE de petite taille et inactifs. **Type 2**: traiter la fracture d'abord puis traiter la lésion causale dans un second temps: c'est le cas notamment de la plus grande partie des KOE et de certains KOA inactifs. **Type 3**: la fracture et la lésion causale sont traitées dans le même temps opératoire: dans ce type, on peut inclure certains KOE étendus du col fémoral, les KOA agressifs et les tumeurs à cellules géantes. Pour les fractures sur **KOE**: dans la littérature, 10 à 30% des KOE guérissent après un ou plusieurs épisodes fracturaire [2]. Cette notion explique le recours au respect initial de la lésion kystique après la première fracture, au membre supérieur et particulièrement l'humérus, l'immobilisation plâtrée et la règle. Au niveau du fémur proximal: la plupart des auteurs recommandent un abord à ciel ouvert avec réduction anatomique et ostéosynthèse interne, le traitement du KOE se fait évidement dans le même temps opératoire. [2, 4], par injection de méthylprédnisone ou Injection intralésionelle de moelle osseuse autologue ou bien chirurgical [2, 4], à ciel ouvert basée sur le curetage de la cavité kystique, qui donne des résultats satisfaisants dans seulement 20 à 40% des cas. C'est pour cette raison, que ce curetage doit être associé à une greffe osseuse adaptée à la plupart des kystes huméraux (Figure 4) et à ceux du membre inférieur (à l'exception du fémur proximal) avec des guérisons autour de 70% - 80%.

**Figure 5:**
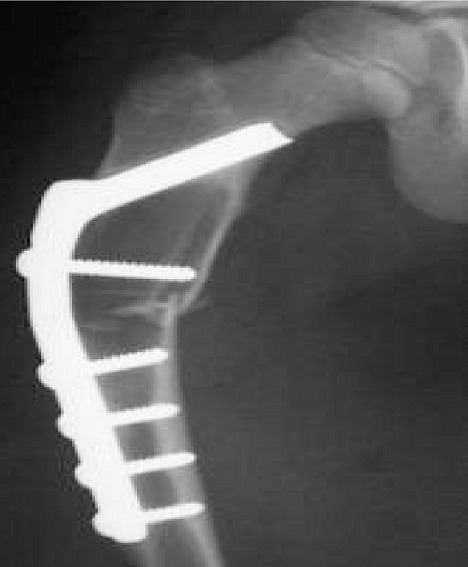
Curetage-greffe osseuse du kyste essentiel huméral et contention par lame plaque

Dans notre série, parmi les 3 atteintes du fémur proximal, 2 ont évoluées favorablement après curetage greffe associé a une lame plaque (Figure 5). Les résultats moyen et mouvais ont été noté après utilisation de broche. Nous estimons que ces 2 résultats sont dus à l'insuffisance de la réduction initiale et à l'utilisation de matériel non rigide, pour l'humérus, nous avons eu 25% de guérison pour les KOE traités chirurgicalement. Car à posteriori, ces kystes étaient très larges et le traitement chirurgical n’était pas agressif. Pour les fractures sur **KOA**: certains nombre de KOA peuvent cacher de véritables tumeurs malignes [2, 4]. Pour ces raisons, la première étape thérapeutique est la biopsie. Celle-ci permet de confirmer le diagnostic et de programmer le geste définitif sur le KOA dans les semaines suivantes. Dans les rares cas associée a des fracture déplacées et/ ou instables, une réduction associée éventuellement à une ostéosynthèse à ciel ouvert permet de stabiliser le foyer et de faire une biopsie exérèse de la lésion tumorale. Pour les fractures sur **FON**.: La plupart de ces tumeurs guérissent après fracture. Nous avons traité 3 fractures sur FNO, toutes traitées orthopédiquement, sauf dans un cas de FNO fémoral distal, ou nous avons réalisé une réduction par manœuvres externes et fixation par ECMES. Le résultat était bon. Pour les fractures sur **CH**: [4], les localisations classiques au niveau des mains ne nécessitent pas de biopsie. En cas de fracture, celle-ci est le plus souvent traitée orthopédiquement. Pour les fractures sur **OSC**: [4], le traitement des fractures est toujours orthopédique. L'exostose est réséquée dans les semaines suivantes si les symptômes reprennent. Pour les fractures des **TCG**: [4]. En pratique, la biopsie peut être nécessaire avant le traitement de la fracture au moindre doute diagnostique. La complexité du traitement de ces tumeurs est majorée par la survenue d'une fracture.

## Conclusion

La pathologie traumatique des membres est extrêmement fréquente chez l′enfant. Le diagnostic est souvent facile, mais il existe de nombreux pièges que le radiologue doit savoir déjouer, en particulier en tenant compte du contexte traumatique et clinique et analysant la structure osseuse sous-jacente afin de ne pas méconnaître une fracture sur os pathologique surtout tumorale bénigne dont la prise en charge adéquate, nécessitant la coopération entre chirurgien, radiologue et anatomopathologiste.
